# Retracted: Gelsolin Restores A*β*-Induced Alterations in Choroid Plexus Epithelium

**DOI:** 10.1155/2021/9815368

**Published:** 2021-04-14

**Authors:** BioMed Research International

*BioMed Research International* has retracted the article titled “Gelsolin Restores A*β*-Induced Alterations in Choroid Plexus Epithelium” [1], due to concerns with duplicated figures as initially raised on PubPeer [2].

Figures 1(a) and 1(b) appear to be identical to Figure 1(a) in [3]. Additionally, in Figure 2, the control and A*β*1-42 panels appear very similar to the control and A*β*1-42 panels in Figure 5(e) [4].

Following an investigation into these concerns, the editorial board has recommended the retraction of the article. The authors do not agree to the retraction.
